# Investigating the Effects of Full-Spectrum LED Lighting on Strawberry Traits Using Correlation Analysis and Time-Series Prediction

**DOI:** 10.3390/plants13020149

**Published:** 2024-01-04

**Authors:** Yuze Lu, Mali Gong, Jing Li, Jianshe Ma

**Affiliations:** 1 Key Laboratory Photonic Control Technology, Ministry of Education, Tsinghua University, Beijing 100083, China; luyz18@mails.tsinghua.edu.cn (Y.L.); gongml@mail.tsinghua.edu.cn (M.G.); 2 International Joint Research Center for Smart Agriculture and Water Security of Yunnan Province, Yunnan Agricultural University, Kunming 650201, China; 3 Tsinghua Shenzhen International Graduate School, Tsinghua University, Shenzhen 518055, China

**Keywords:** light quality, strawberry traits, correlation coefficient, CEEDMAN method, informer network

## Abstract

In crop cultivation, particularly in controlled environmental agriculture, light quality is one of the most critical factors affecting crop growth and harvest. Many scholars have studied the effects of light quality on strawberry traits, but they have used relatively simple light components and considered only a small number of light qualities and traits in each experiment, and the results were not complete or objective. In order to comprehensively investigate the effects of different light qualities from 350 nm to 1000 nm on strawberry traits to better predict the future growth trend of strawberries under different light qualities, we proposed a new approach. We introduced Spearman’s rank correlation coefficient to handle complex light quality variations and multiple traits, preprocessed the cultivation data through the CEEDMAN method, and predicted them using the Informer network. We took 500 strawberry plants as samples and cultivated them in 72 groups of dynamically changing light qualities. Then, we recorded the growth changes and formed training and testing sets. Finally, we discussed the correlation between light quality and plant trait changes in consistency with current studies, and the proposed prediction model achieved the best performance in the prediction task of nine plant traits compared with the comparison models. Thus, the validity of the proposed method and model was demonstrated.

## 1. Introduction

The first applications of light-emitting diodes (LEDs) in the 1980s propelled the development of plant lighting for controlled-environment agriculture (CEA), enabling cultivation in various extreme environments [1,2]. With diverse and manually adjustable light sources, LED lighting can accommodate different plant needs and substantially enhance planting efficiency.

Extensive research has examined the effects of light quality (spectral composition) on plants. However, apart from providing energy for plant growth and reproduction, light exposure also influences secondary metabolites and other factors, which play a regulatory role in plants, significantly impacting their traits. According to Davis et al. [3], the effective radiation range for photosynthesis in plants is 400–700 nm. In addition to various phytochromes with distinct absorption peaks at 600–700 nm and 400–500 nm, other wavelength ranges still regulate plant traits through different mechanisms [4,5]. In terms of light quality effects on plant traits, Samuolienė et al. [6] demonstrated that a 7:1 red-to-blue spectrum promoted larger strawberry fruits and higher sugar content, but at the same time, inhibited flowering [7]. Piovene et al. [8] experimentally observed that the best strawberry growth and fruit quality was achieved with a red-to-blue light ratio of 7:10, but the best strawberry growth rate was obtained with a 34% blue light content [9]. In addition, red and blue light can have a significant effect on the roots, stems, and leaves of plants [10,11,12]. Furthermore, other bands of the spectrum, such as yellow–green light, can also have effects on crop morphology [13,14,15].

In addition to the extensively studied spectrum within the visible light range of 400–700 nm, other spectral regions, such as ultraviolet (UV) wavelengths, deep red light, and infrared (IR) wavelengths, also have effects on plant morphology. UV-A radiation has been found to cause inhibition of PSII activity [16]; however, it could actually stimulate photosynthesis at lower doses [17]. Furthermore, moderate UV exposure before harvest has been shown to improve the quality and extend the storage time of fruits and vegetables [18,19]. Without taking into account additional factors, the effects of UV on plants are still inconclusive [20]. Moreover, Dubois et al. [21] demonstrated that far-red light significantly affects plant height under shaded conditions. Abundant far-red light has been found to result in thinner and narrower leaves [22,23], but it promotes plant radiation use efficiency [24,25].

In this area of research, it appears that researchers have conducted substantial investigations into the spectral conditioning of plant traits, yet limitations and challenges remain pronounced given current experimental methods. This is due to the large spectral range that has to be researched and the possibility that each spectral interval may have an impact on plant morphology. As cited above, current relevant studies primarily consisted of straightforward group experiments and combinations of one to three colors of light sources to determine various crop trait performances. Alternatively, a particular trait is specified, and several light sources are chosen for tests to investigate their effects. This ignores the fact that the spectrum is continuous and that the combination of multiple spectral bands has a joint effect on plants. What is more, the same light quality can have different effects on different plant species, and even for the same crop, varying light intensities, temperatures, and humidity conditions can lead to different regulatory outcomes [26].

To comprehensively consider a broad spectrum and multiple traits, the problem of large amounts of data and the complex correlation between data needs to be solved. As far as we know, there are still no studies that utilize deep learning methods to consider the effects of light quality on plant traits from a broad-spectrum perspective. Thus, solutions based on neural networks have been introduced in this research. Informer was proposed [27], which was improved based on Transformer [28], to reduce computational complexity and avoid cumulative error diffusion when outputting long sequence data. Indeed, the Informer network’s architecture enables the incorporation of complex, time-series information regarding broad-spectrum changes into the model. It efficiently outputs changes in plant traits over the medium-to-long term in a single pass. Since trait predictions at each moment are influenced by the preceding moment, the Informer network, with its reduced cumulative error diffusion, is highly suitable for this study. This design ensures accurate and reliable predictions of plant traits, making it well-suited for the analysis of light quality effects on plants in a comprehensive manner. For example, Lu et al. [29] combined reinforcement learning with Informer to predict the fruit and stolon harvest of strawberry and to provide real-time feedback on the optimal growth environment. Wu et al. [30] utilized the Informer network model to make a prediction of photovoltaic power by taking complex weather variability factors into account. However, Informer-related models are still not applied much in agriculture, and their modeling advantages need to be exploited urgently.

In this research, we grouped the strawberry samples and exposed them to varying LED light qualities. Each group experienced a distinct sequence of light qualities over time. This protocol enabled us to amass an ample training dataset that encompassed variations in LED light quality and their corresponding shifts in traits. The correlation coefficient was introduced to measure the relationship between the light quality and various traits of strawberry. Based on the correlation coefficients, we manually intervened in the prediction network to improve the prediction performance. In order to avoid the model overfitting problem due to the possible insufficient data, we utilized the CEEDMAN method to decompose the original data, which increased the diversity and complexity of the data. Furthermore, we introduced random fluctuations in the LED light quality to compare the projected trait changes by the Informer model with the empirically observed ones. This comparative analysis served to authenticate the efficacy and precision of our model. With this method, in just one experiment, we established a connection between multiple combinations of light quality and changes in multiple plant traits, and based on this, we readily regulated the optimal light regimen for different growth periods.

## 2. Experiments and Results

### 2.1. Correlation between Light Qualities and Traits

The study aimed to uncover the relationship between combinations of light qualities and sample traits, not only examining the impact of individual light bands but also the effects of multi-band light quality combinations on sample traits. A substantial volume of data was generated on light quality and plant samples during the experiment. Spearman’s rank correlation coefficient was employed to examine the influence of each light quality (or combination of light qualities) on multiple plant traits. Due to the abundance of input variables, the correlation between independent variables created a challenge of collinearity. As the number of input variables increased, the demand for model training capability heightened, leading to potential overburdening of the model during training and testing. Consequently, correlation analysis was utilized to initially assess the influence of each light quality (or combination of light qualities), and subsequently, factors displaying high correlation were filtered out to alleviate the computational strain induced by data collinearity.

According to the spectral intervals, we divided the spectra of illumination into nine intervals: UVA (350–380 nm), violet (380–400 nm), indigo (400–450 nm), blue (450–495 nm), green (495–570 nm), yellow (570–590 nm), orange (590–620 nm), red (620–750 nm), and near-infrared (750–1000 nm). The corresponding traits and basic information of the plant samples are shown in Table 1.

**Figure 1:**
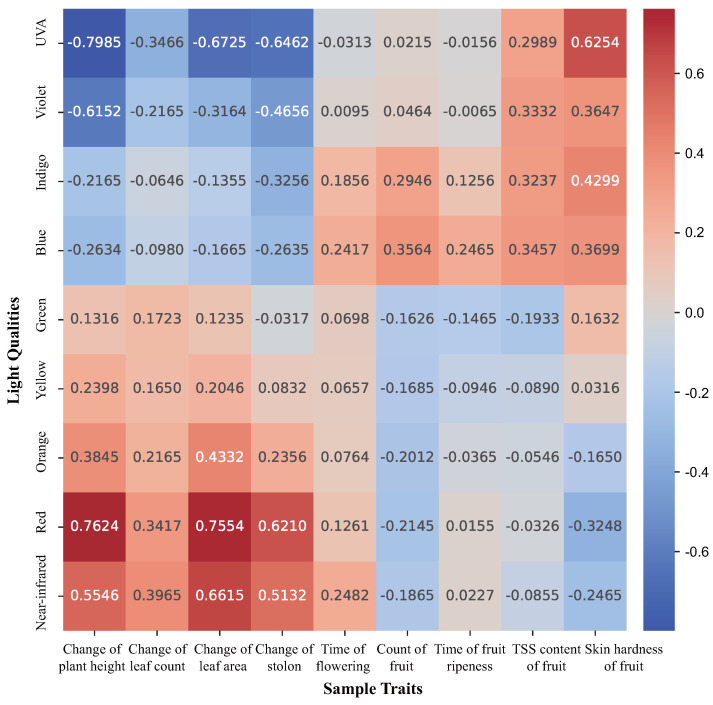
Spearman’s rank correlation heat map between the light qualities and sample traits.

We analyzed the correlation between the collected large-scale trait data and light quality and plotted the correlation coefficients in a heat map as shown in Figure 1.

As observed in the heat map, a significant disparity existed in the impacts of various light qualities on diverse plant traits. In general, light qualities characterized by longer wavelength bands facilitated the growth and proliferation of the plant body, whereas those with shorter wavelength bands enhanced the fruit quality of the plant body. In comparison with prior studies, our experimental illumination spectrum was continuous and comprehensive, reflecting the continuous growth state of the plant, which aligned more closely with actual growth conditions. Furthermore, the data obtained from our experiments represented the combined effect of multiple light qualities. Taking red light (620–750 nm) as an example, the investigation into the influence of red light on plant traits involved the simultaneous presence of other wavelengths of irradiated light in all experimental groups. The findings were not simply derived from the amalgamation of red light, blue light, green light, and so forth, but rather from a complex interplay of various light qualities.

Certain correlation coefficients with very small absolute values were attributed to the insignificant impact of light quality combinations on specific traits or stemmed from the nonlinearity of light quality effects. For instance, a small quantity of the violet light component was found to accelerate the ripening time of fruits; however, as the intensity increased, it began to hinder the ripening process instead.

We took Spearman’s rank correlation into account as part of the input to the subsequent prediction network and adjusted the influence weights of each light quality combination based on the magnitude of the correlation coefficient. This step enhanced the accuracy of the prediction network.

### 2.2. Prediction of Informer Network

Drawing from the light quality combinations and strawberry (Fragaria × ananassa Duch. “Zhang Ji”) sample traits with significant correlation coefficients, we artificially adjusted the parameter weights of the input network model using the initial inputs. In the validation experiments within this section, we inputted the data, processed by the CEEDMAN method, into the Informer network for prediction, allowing for a comparison of the predictive effects of various preprocessing methods.

The comparative experiments were divided into four groups. The first group involved the direct input of the most original unprepared data into the Transformer for prediction. The second group entailed the direct input of the most original unprepared data into Informer for prediction. The third group included data processed by the CEEDMAN method and was input into Informer for prediction. Lastly, the fourth group encompassed data preprocessed by the CEEDMAN method and adjusted by correlation coefficient weights before being input into Informer for prediction. Throughout all the experiments, we not only verified the superiority of Informer in this task but also confirmed the enhancement of the model’s prediction ability through the CEEDMAN method preprocessing.

**Figure 2:**
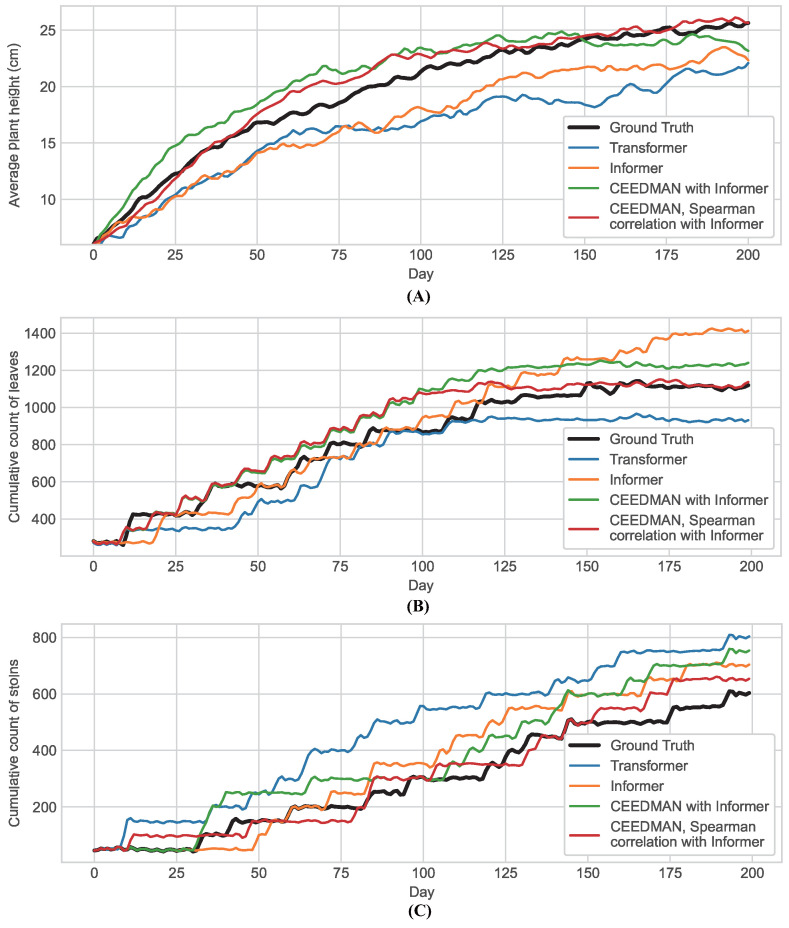
Prediction curves of comparative experiments. (**A**) The change of plant height: shown in the curves is the average plant height of the sample. (**B**) The change of leaf count: shown in the curves is the cumulative count of the leaves. (**C**) The change of stolons: shown in the curves is the cumulative count of the stolons.

**Figure 3:**
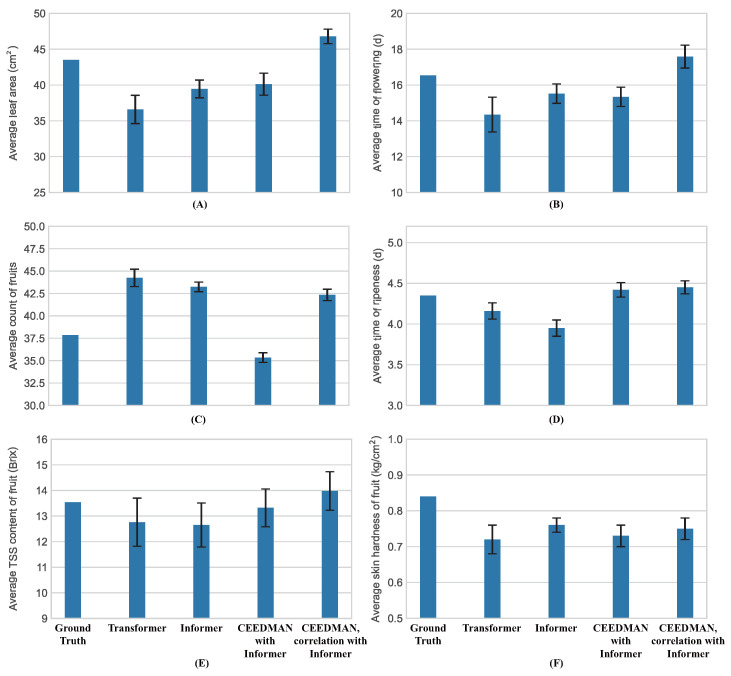
Prediction results of compared methods. (**A**) Average leaf area. (**B**) Average time of flowering. (**C**) Average count of fruits. (**D**) Average time of ripeness. (**E**) Average TSS content of fruit. (**F**) Average skin hardness of fruit.

The performance of all considered traits of strawberry samples in the four prediction models described above was indicated by the corresponding expressions in Figure 2. Changes in leaf area, time of flowering, fruit count, time of fruit ripeness, TSS content, and skin hardness, which constituted the six traits’ data, were non-continuous and, hence, could not be represented by changed curves. Therefore, these prediction models were employed to predict the data collected at each instance, and their predictions were compared with the actual data to assess the performance of each set of predictions. The histogram of their prediction results is shown in Figure 3.

From Figure 2 and Figure 3, it was observed that Informer processed with CEEDMAN and Spearman’s rank correlation exhibited overall superior prediction results. Compared with Transformer, Informer without preprocessing also showed significantly improved prediction results, suggesting that Informer does indeed possess an advantage in this type of task. To provide a more precise representation of the prediction effect, the $R^{2}$ and SEP measures were used for quantitative evaluation, which are shown in Table 2.

The CEEDMAN and Spearman’s rank correlation preprocessed Informer methods accounted for 10 of the 18 best indicators and ranked among the top of all methods in terms of other metric scores.

## 3. Discussion

Our experiments were designed to provide diverse growing environments for strawberry samples using multiple sets of full-spectrum illumination light. This approach allowed us to investigate the effects of light quality in each wavelength band on specific plant traits. The conclusions were then integrated with the prediction model to enhance the performance of the Informer and improve the model’s prediction results. In comparison with previous studies, this was the first time that we investigated the traits of samples under full-spectrum conditions. In contrast, some experiments only combined two or three spectra, which overlooked the comprehensive effects of multiple combined light qualities.

Additionally, the conclusions drawn in our study were compared with those of current studies. In our study, the UVA and violet wavelengths inhibited the growth of sample plant height, leaves, and stolons. As demonstrated by current research, these short wavelength light qualities suppressed the expansion of leaf area and delayed the accumulation of biomass, which was proven in many studies [31,32,33]. This was because short-wavelength spectra inhibited the secretion of plant gibberellins and auxins. Additionally, it was found that UV treatments delayed the loss of firmness in various fruits [34,35], and that UVA and short wavelengths promoted sugar accumulation and fruit skin firmness in strawberry fruit [36,37]. This was also commonly employed in post-harvest fruit treatments. These studies believed that UVA inhibited ethylene synthesis in fruits, thereby reducing the fruit’s ripening rate and decay susceptibility. Furthermore, it was hypothesized that UVA might have triggered the accumulation of secondary metabolites in fruits. Some polyphenolic compounds and carotenoids, for instance, may have accumulated under UVA exposure. These compounds not only enhanced the fruit’s antioxidant capacity but also contributed to increased fruit firmness and flavor enhancement.

In the current work, research on UVA is far less abundant compared with studies on UVB, despite the fact that UVA intensity in solar radiation is 10–100 times higher than UVB, and studies on the effects of UVA on crops still yield conflicting results [38,39]. From a biological and photoreceptor perspective, some studies have demonstrated that UVA can influence the accumulation of substances, such as flavonoids, anthocyanins, and total phenolics in leaves [40,41,42]. Furthermore, a study assessed the impact of supplemental UVA radiation on various antioxidant levels in the leaves of Rosa hybrida and Fuchsia hybrida, revealing that UVA induced a slight increase in chlorophyll a and b; carotenoids, including lutein, zeaxanthin, and β -carotene levels; and a substantial increase in flavonols, quercetin, kaempferol, and their derivatives [41]. However, none of these studies have been able to provide a comprehensive and detailed explanation of the underlying mechanisms. In the case of Arabidopsis thaliana, it has been suggested that UVA reception may involve three known photoreceptors: (1) phytochromes (PHY) for far-red and red light, (2) cryptochromes (CRYs) for UVA/blue light, and (3) phototropins (PHOT) for UVA light [20,43]. Once UVA light is perceived, these UVA-specific photoreceptors may interact with constitutively photomorphogenic 1 (COP1) and hypocotyl 5 (HY5) in a manner similar to UV-resistant locus 8 (UVR8), subsequently modulating secondary metabolism in plants. From this perspective, in our study, we deduce that UVA radiation stimulates the synthesis of secondary metabolites in strawberry samples, increasing the accumulation of organic compounds in the fruits, enhancing antioxidant properties, and thereby improving fruit hardness. Zhang et al. [33] postulated that the morphogenic effects of UV-A exclusion might be mediated through cryptochrome or photoreceptor receptors, which, in turn, could affect gibberellin accumulation in internodes, influencing plant height and leaf area. The specific impact is dependent on factors, such as temperature, humidity, and light intensity in which the plants are grown [20]. In our experiment, UVA resulted in a compact phenotype in the samples.

Blue light is one of the crucial light qualities in plant photosynthesis, and according to our experiments, light qualities in this spectral band played a mildly inhibitory role in morphological growth and asexual reproduction. However, it also had a promotional effect on sexual proliferation and fruit quality of plants [7,44,45]. Currently, there were many studies on the blue light quality band, and their perspectives differed. For instance, Xu et al. [46] observed a significant increase in strawberry leaf area and leaf crown with increasing LED blue light intensity. However, we and other studies [6,47] have demonstrated the inhibitory effect of blue light on strawberry stem and height. This discrepancy might be attributed to differences arising from the use of different strawberry varieties and light quality combinations in the design of the experiments. Overall, the effects of this light quality spectral band on strawberry traits were, in general, in agreement with our conclusions.

From a physiological perspective, secondary metabolites, such as flavonoids and quinones, protect plants from oxidative damage induced by the scavenging of free radicals [48]. The most prevalent secondary metabolites in strawberry fruit are flavonoids, including anthocyanins [49]. These flavonoid compounds primarily shield plants from damage caused by short-wavelength spectra [50], among which anthocyanins protect plants from harm by blue and green light. Therefore, when the intensity of blue light is increased, it can elevate the content of these flavonoid compounds, enhancing fruit quality (color, antioxidant properties, organic compounds, etc.). In terms of plant photomorphogenesis, Kalaitzoglou et al. [51] confirmed that with the increase in blue light intensity, both leaf area and plant height of the plant body significantly decreased. They speculated that this was also induced by CRYs because CRYs are the primary photoreceptors that inhibit the elongation of the hypocotyl and epicotyl [52], and blue light stimulates the secretion of CRYs. Additionally, blue light can alter the expression of sensory proteins in strawberries, promoting the expression of transcription factors, such as BBX and FaBBX29 [53], thereby significantly enhancing strawberry flowering and fruiting. However, excessively strong blue light intensity may inhibit the total photosynthetic capacity of the plant body by promoting a compact phenotype [51], which could potentially affect fruit size. Thus, the impact of blue light quality on strawberries is not entirely positive.

The effect of light quality on strawberry traits underwent significant changes with increasing wavelength. Among these, yellow–green light, which is less involved in photosynthesis, still had highly controversial effects on traits. Some studies indicated that yellow–green light quality favored plant stem and leaf growth [54,55], but Posada et al. [56] argued the opposite. They discovered that green light modified the distribution pattern of dry matter in plant organs, thus inhibiting strawberry growth. However, none provided a clear biological explanation. In our study, yellow–green light slightly promoted strawberry plant height, leaf, and stolon growth, while also slightly inhibiting sexual proliferation and fruiting. The correlation coefficient between light quality and traits was low. We hypothesized that this was because the yellow–green light spectrum falls between the blue and red light ranges, and it combines the effects of both blue and red light. This is also why the impact of yellow–green light on plants has been a subject of ongoing debate. In our future research, we will conduct a detailed exploration of its effects.

In the field of physiology, specific yellow–green light receptors have not yet been identified in higher plants [57,58]. Therefore, some studies suggest that plants perceive yellow–green light through the residual sensitivity of blue and red light receptors [59], and these receptors have low sensitivity to the main wavelengths of yellow–green light. Consequently, this inevitably leads to the conclusion that the impact of yellow–green light on plants is less significant compared to other light qualities [60]. These physiological mechanisms are in line with our experimental results.

Light quality in the red-to-near-infrared range was the most crucial part involved in plant photosynthesis and bioregulation and had been the subject of extensive study. Studies generally demonstrated that red light (near-infrared light) had a significant stimulating effect on the leaves, stolons, and plant height of strawberry plant bodies [6,56,61,62], leading to an increase in the dry weight of the aboveground part of the plant body [61]. This was because an excess of gibberellins was secreted within the strawberry plants. However, because this light quality part promoted the growth and asexual reproduction of the plant body, it had a negative impact on sexual reproduction and fruit development, resulting in issues such as a reduction in the number of flower clusters [44] and a decrease in fruit size [6]. In our experiments, we also found that long-wave light quality had a significant promoting effect on the plant height, leaf, and stolon growth of the samples (positive correlation was evident). Additionally, it had a slight inhibitory effect on flowering, ripeness, TSS content, and fruit hardness of the samples (negative correlation and the absolute value was relatively small).

From a mechanistic perspective, unlike blue light responses primarily mediated by CRYs and PHOT [63], the regulation of red and near-infrared light is closely associated with plant photoreceptors, particularly phytochromes (phyA–phyE) [64]. In terms of plant physiological regulation, research [65] demonstrated that phyA significantly induces plant germination, which is specifically stimulated by red and near-infrared light. Additionally, phytochromes can stimulate the growth of the hypocotyl [66], expansion of cotyledons [67], elongation of petioles [68], and enlargement of leaf area [69], enhancing a plant’s ability to overcome gravity and resulting in increased plant size [70]. However, an excess of red light often leads to excessive elongation of plant roots, stems, and leaves, thereby reducing the nutrient availability required for flowering and fruiting.

Our experimental results aligned closely with the findings of the current study, and our experimental results are well confirmed physiologically as well. Moreover, the enhanced predictive accuracy of the prediction model, attributed to the “light quality-trait” correlation coefficient, further substantiated the robustness of our conclusions. Notably, in this study, all the light qualities and traits were obtained in one experiment, significantly reducing the number and cost of experiments. The Informer model, preprocessed by the CEEDMAN method and Spearman’s rank correlation coefficient, made excellent predictions for all nine sample traits compared to the other three control models, and the overall performance was the best.

## 4. Materials and Methods

### 4.1. Processing of Strawberry Samples

Strawberry was chosen as the experimental sample because it is a commercial crop with multiple growth stages and multiple traits in the plant, flowers, and fruits. A total of 500 strawberry plants were sampled in the experiment, and they were grown from seedlings with three to five compound leaves in soil culture (peat soil, perlite, vermiculite, charred rice husk, etc.). The strawberry samples were drip irrigated using diluted Yamazaki nutrient solution [71] with an electrical conductivity (EC value) of 800 μ s/cm. The automated drip irrigation system (Zeego, ZG-2100, Guangzhou, China) was timed daily to maintain the soil moisture level of about 70%. The day and night temperature of the incubation room was 15–25 °C, air humidity was 60–75%, and CO_2_ level was 800–1000 ppm.

**Figure 4:**
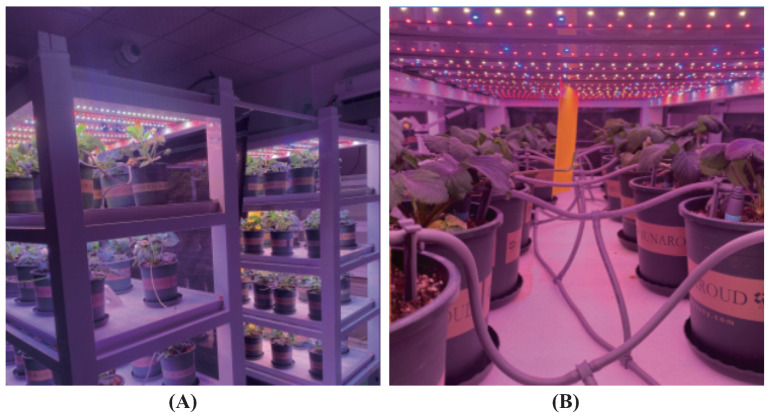
Cultivation and grouping of strawberry samples. (**A**) Sample incubation racks. (**B**) Lighting, irrigation, and grouping of samples.

To mitigate the potential introduction of random errors resulting from identical planting times, a strategic approach was adopted. The strawberry samples were organized into 10 distinct groups, each group initiated at varying planting times with intervals spanning roughly 10–15 days. Also, each group underwent a 200-day cultivation period. The cultivation environment of the sample is demonstrated in Figure 4.

#### 4.1.1. Light Quality Processing of Subgroups

**Figure 5:**
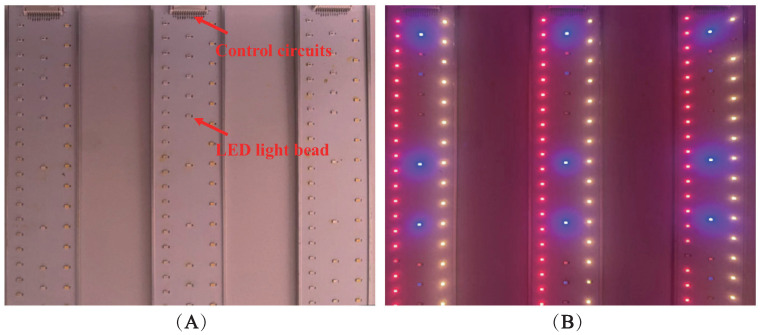
Layout of light-emitting diodes (LEDs) on the illumination boards. (**A**) Distribution of parts when not in operation. (**B**) Illumination status when in operation.

**Figure 6:**
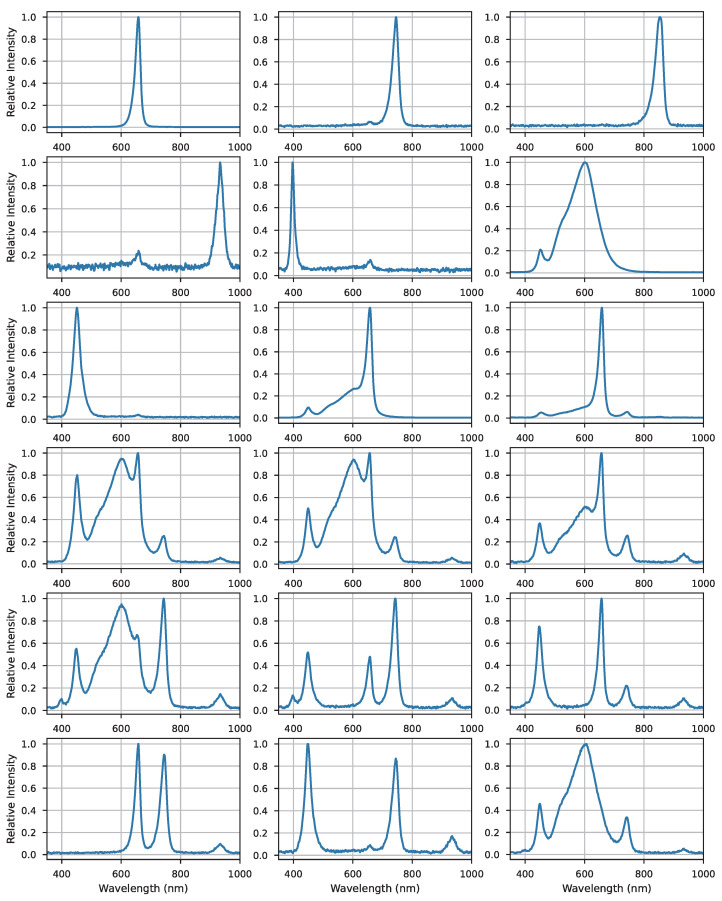
Some of the light quality combination curves.

The illumination board in the experiment was designed and fabricated by us, and the lighting source was LED (JOMHYM, JH-STX3535, Dongguan, China). Multiple wavelengths of LEDs were soldered to the control circuit board to ensure random combination of light sources and uniformity of light illumination. The illumination board has seven single-source LEDs covering the UVA (350 nm) to near-infrared shortwave (1000 nm) range. In our experiments, we independently modulated the switching and intensity of each LED, resulting in varying combinations of total light quality. Furthermore, the regulation of lighting for different experimental groups was randomized, with alterations in light quality combinations occurring every 2 to 3 days. The LEDs worked 16 h a day (4:00 a.m. to 8:00 p.m.). This approach allowed ample time for the strawberry samples’ traits to evolve. We changed a total of 72 different combinations of light qualities and employed a portable spectrometer (StellarNet, BLK-CXR-SR, Tampa, FL, USA) to detect and record them throughout the experimentation process. The layout of the lighting board is depicted in Figure 5, and some light quality combinations are shown in Figure 6.

According to Zheng et al. [72], we maintained the total light intensity for each combination of light quality within the range of 250 to 300 μ mol/(m^2^·s) in our experiment. This range was chosen due to its optimal suitability for promoting the growth of various aspects of strawberry plants, including branches, stolons, flowers, and fruits, cultivated under artificial lighting conditions.

#### 4.1.2. Acquisition of Strawberry Traits

In this experiment, according to Guiamba et al. [73] and Matysiak et al. [74], we assessed 9 specific strawberry traits, including the changes of plant height, leaf count, leaf area and stolon, time of flowering, count of fruit, time of fruit ripeness, total soluble solid (TSS) content of fruit, and skin hardness of fruit.

During the initial planting stage, each strawberry seedling sample started at a similar developmental stage. Consequently, we assumed uniformity in the growth states of the samples within the same environmental conditions. Thus, it could be inferred that the growth states of strawberry samples in each subgroup were homogenous. Hence, when assessing the sample trait parameters, we employed a random selection process to choose 20 samples from each group, which comprised a total of 50 samples. This selection method effectively represented the collective growth state of the samples within that specific group. The changes in plant height and leaf area were measured by dial calipers, the TSS content of fruit was detected by an IR Brix meter (ATAGO, PAL-HIKARi 4, Tokyo, Japan), and the skin hardness of fruit was measured by a fruit hardness tester (Ruinong, GY-3, Zhengzhou, China).

### 4.2. Pre-Processing of Data

The LED light quality and the trait information of the strawberry samples are multidimensional time-series data, with the former being the independent variable and the latter the dependent variable. LED light quality information was low-frequency variable data, changing only once every 2 to 3 days. This is because plant samples are only exposed to light for a sufficient amount of time before trait changes occur, and this process is relatively slow. If the light quality changed too often, when the sample traits began to produce changes, and the LED light quality had already changed, it was impossible to determine which light quality caused the change in the sample traits.

Trait parameters were measured every half day because trait changes are very rapid during the peak growth periods of strawberry plants (e.g., flowering, fruiting, ripening, etc.). When the strawberry fruits were ripe, the strawberries were picked and measured for TSS content and skin hardness. When recording parameters, the rows of data were labeled with time, and the columns were labeled with the relative intensities of the different bands of LEDs and the corresponding plant trait parameters. Because there was a delay between trait changes and light quality changes, the trait parameters were shifted up one day when the data were recorded.

The data were recorded with a minimum time interval of half a day (12 h) and a total time duration of 200 days with 10 groups of data; therefore, the total number of rows in the dataset was 4000. The training and testing sets were divided in 8:2. However, the volume of this dataset was too small and could easily be underfitted in a high-complexity network such as Informer. Moreover, the purpose of our study was to explore the effect of the light quality of LEDs on the traits produced by strawberry samples, and ways should be found to expose as many features of the different light qualities of LEDs as possible.

#### 4.2.1. CEEDMAN Decomposition Method

Inspired by Torres et al. and Xu et al. [46,75], we introduced the complete ensemble empirical mode decomposition with adaptive noise (CEEDMAN) method into the pre-processing of data. It is an improvement and extension of empirical mode decomposition (EMD) [76], which aims to better deal with noise in signals and extract the intrinsic structure of signals. It can decompose a complex signal into a finite number of intrinsic mode functions (IMFs), each of which preserves the local feature signals of the original signal on different time scales, its most important feature. The CEEMDAN method involves the following steps.

The original signal might contain a certain amount of Gaussian noise. Determine the initial $IMF_{1}$ of the original signal using the ensemble average of the total $IMF_{1}$ corresponding to *N* trials,

(1) $$\hat{IMF_{1}\left(t\right)}=\frac{1}{N}\sum_{n=1}^{N}E_{1}\left(X\left(t\right)-\varepsilon \omega_{n}\left(t\right)\right)$$

where $\hat{IMF_{1}\left(t\right)}$ was the first EMD component of input signals, $\varepsilon \omega_{n}\left(t\right)$ was the Gaussian white noise with N(0,1) . $E_{1}\left(\cdot \right)$ was the *i*th IMF component and *N* is implementation number. The first signal residual $r_{1}$ was calculated by $r_{1}=X-\hat{IMF_{1}}$ .

Similar to Equation (1), the second EMD component of input signals $\hat{IMF_{2}\left(t\right)}$ was calculated by Equation (2):

(2) $$\hat{IMF_{2}\left(t\right)}=\frac{1}{N}\sum_{n=1}^{N}E_{1}\left(r_{1}\left(t\right)-\varepsilon E_{1}\left(\omega_{n}\left(t\right)\right)\right)$$

Likewise, we can obtain the equations for the $IMF_{k+1}$ th and *k*th residual values as in Equations (3) and (4):

(3) $$\hat{IMF_{k+1}\left(t\right)}=\frac{1}{N}\sum_{n=1}^{N}E_{1}\left(r_{k}\left(t\right)-\varepsilon E_{k}\left(\omega_{n}\left(t\right)\right)\right)$$

(4) $$r_{k}=r_{k-1}-\hat{IMF_{k}}$$

The Equations (1)–(4) were repeated until the extreme values of the last residual value were less than two.

(5) $$R=X-\sum_{k=1}^{K}\hat{IMF_{k}}$$

where *R* was the last residual value, and the IMF number was *K*. The last decomposition result was calculated by Equation (6). Finally, this method ensured proper signal decomposition and reconstruction.

(6) $$X\left(t\right)=\sum_{k=1}^{K}\hat{IMF_{k}\left(t\right)}+R\left(t\right)$$

#### 4.2.2. Decomposition of Original Input Signals

**Figure 7:**
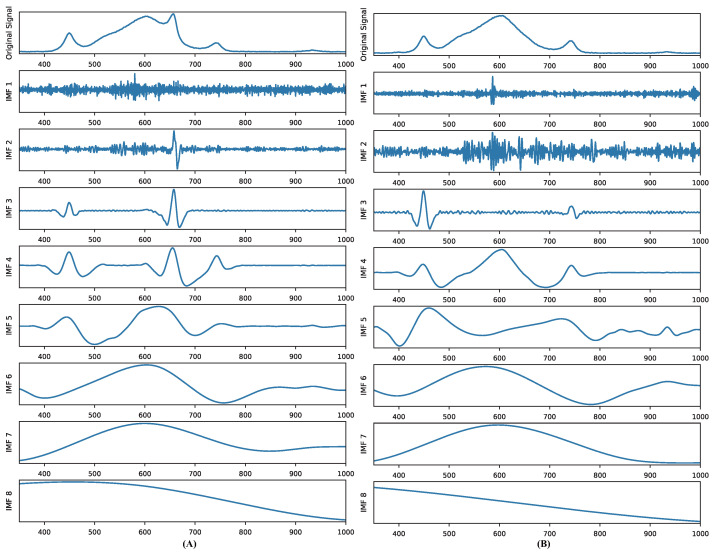
Two of the original light-emitting diode (LED) spectra and their decomposed intrinsic mode function (IMF) curves.

Prior to feeding the data into the time-series network model, we utilized the CEEDMAN method to decompose the spectral data representing LED light quality, yielding 8 IMF components. This approach served a dual purpose: Firstly, it augmented the dataset’s volume to align with the complex structure of the Informer model, mitigating the risk of underfitting. Secondly, it facilitated a clearer observation and analysis of the distinct frequency components’ characteristics. Additionally, this decomposition aided the subsequent model in capturing local variations and spectral features with greater precision, free from interference caused by the overall signal shape. Two of the original LED spectral information and their decomposed IMFs are presented in Figure 7.

The light source band utilized in the experiment spanned from 350 nm to 1000 nm, with a spectrometer acquisition interval of 0.5 nm. The original signal’s shape before decomposition was 1 × 1300. Following decomposition, the signal became multidimensional, with a shape of 8 × 1300. Subsequent dimensional reduction resulted in a new shape of 1 × 10,400.

### 4.3. Informer Model

The Informer was improved based on Transformer. Compared to typical RNN time-series networks, the multi-head self-attention mechanism in Transformer allowed the model to learn different focuses of attention and feature representations at the same time. However, each head in the multi-head self-attention mechanism had a set of attention weight matrices, which increased the complexity of the model; especially for long-term prediction, it put a lot of computational pressure on the hardware [77]. The typical self-attention [28] was based on a tuple input method, including the Key, Value, and Query. They were connected in the scaled dot product as in Equation (7):

(7) $$A\left(Q,K,V\right)=softmax\left(\frac{QK^{⊤}}{\sqrt{d}}\right)V$$

where $Q\in R^{L_{Q}\times d}$ , $K\in R^{L_{K}\times d}$ , $V\in R^{L_{V}\times d}$ and *d* presented the dimension of input data. The *i*th query’s attention was defined as a kernel smoother in a probability form as in Equation (8):

(8) $$A\left(q_{i},K,V\right)=\sum_{j}\frac{k(q_{i},k_{j})}{\sum_{l}k\left(q_{i},k_{l}\right)}v_{j}=E_{p\left(k_{j}|q_{i}\right)}\left[v_{j}\right]$$

where $p\left(k_{j}|q_{i}\right)=k\left(q_{i},k_{j}\right)/\sum_{l}\left(q_{i},k_{l}\right)\;$ and $k\left(q_{i},k_{j}\right)=\exp\left(q_{i}k_{j}^{T}/\sqrt{d}\;\right)$ , requiring a computational complexity of $O(L_{Q}L_{K})$ , which was of squared magnitude, and which was the main drawback in improving the predictive power.

In order to reduce the computational cost, Informer introduced the distillation self-attention mechanism, which calculated the activity of each query using the Kullback–Leibler divergence [78], and eliminated the “Lazy” queries that have very little contribution in the typical self-attention mechanism, which decreased the waste of computational resources caused by the redundant parameter storage. The ProbSparse attention mechanism was improved from Equation (7):

(9) $$A\left(Q,K,V\right)=softmax\left(\frac{\bar{Q}K^{⊤}}{\sqrt{d}}\right)V$$

where $\bar{Q}$ was a sparse matrix of the same size of *q* and it only contains Top-u “Active” queries, $u=c\cdot lnL_{Q}$ and *c* was a customized hyper-parameter. Naturally, the computational complexity was reduced to $O(L_{K}lnL_{Q})$ .

In addition, generative reasoning was utilized in the decoder to cope with the challenge of slow long-term prediction of typical decoder structures. A known sequence was used as a start sequence to guide the decoder to generate the target sequence in one step, eliminating the need for a dynamic decoding process and improving output efficiency. The decoder was provided as follows:

(10) $$X_{de}^{t}=Concat\left(X_{known}^{t},X_{0}^{t}\right)\in R^{\left(L_{known}+L_{y}\right)\times d_{model}}$$

where $X_{known}^{t}\in R^{L_{known}\times d_{model}}$ presented the known guiding sequence and $X_{0}^{t}\in R^{L_{y}\times d_{model}}$ was the future sequence to be predicted.

**Figure 8:**
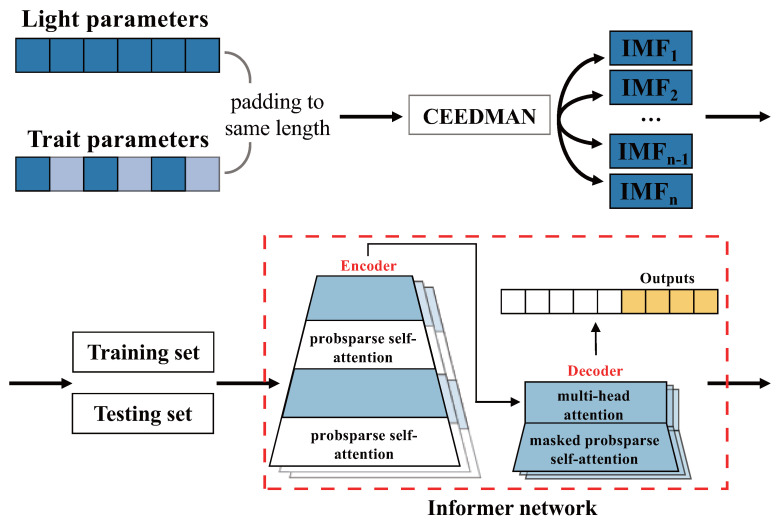
The full complete ensemble empirical mode decomposition with adaptive noise (CEEDMAN) and Informer data processing flow.

In the validation experiment, the whole data processing flow is shown in Figure 8. The key hyper-parameters of the trained model are indicated in Table 3.

### 4.4. Evaluation of Correlation

The combinations of light quality and the trait changes of the samples in the experiment were complex. Moreover, a certain trait of a sample could be affected by a certain spectral band or a combination of spectral bands. In addition, when a certain light quality intensity went from low to high, the effect on the samples might be nonlinear, such as the complex effect of UVA [79] and the “red light syndrome” [80]. In order to fully analyze the relationship between the effects of complex light-quality components and plant traits, we introduced Spearman’s rank correlation coefficient. It is a non-parametric statistic that measures the correlation between variables by converting the raw data to rank order (sorted by size) and, therefore, does not require the data to be normally distributed. It is suitable for situations where the relationship between variables is monotonic but not necessarily linear, or where the raw data do not satisfy the requirements for linear correlation. It is expressed as Equation (11).

(11) $$\rho =1-\frac{6\sum d_{i}^{2}}{n(n^{2}-1)}$$

where ρ denotes Spearman’s rank correlation coefficient, $d_{i}$ denotes the rank difference between the two sets of data, and *n* is the volume of the samples.

### 4.5. Evaluation and Indicators

The evaluation metrics for subsequent prediction models were $R^{2}$ , which measured the correlation between the ground truth and predicted values, and SEP , which represented the percent standard error of the prediction. Their formulas are as follows:

(12) $$R^{2}=1-\frac{SS_{res}}{SS_{tot}}=1-\frac{\sum_{i=1}^{n}{\left(X_{truth,i}-X_{pred,i}\right)}^{2}}{\sum_{i=1}^{n}{\left(X_{truth,i}-{\bar{X}}_{truth,i}\right)}^{2}}$$

(13) $$SEP\left(\%\right)=\frac{100}{{\bar{X}}_{truth,i}}\sqrt{\frac{1}{N}\sum_{i=1}^{n}{\left(X_{truth,i}-X_{pred,i}\right)}^{2}}$$

where $SS_{res}$ is the sum of squares of residuals, which measures the variation that remains unexplained after performing the regression; $SS_{tot}$ is the total sum of squares, which represents the total variance in the dependent variable. $X_{truth,i}$ is the ground truth collected by measuring equipment, $X_{pred,i}$ is the predicted results output by our model, and ${\bar{X}}_{truth,i}$ is the mean value of the ground truth.

## 5. Conclusions

In controlled-environment agriculture, light is one of the key factors affecting crop growth and fruit harvest. Each spectral band of light could positively or negatively affect a certain trait of the plant. Furthermore, light, in addition to providing the energy for photosynthesis in plants, stimulates various receptors, resulting in the production of multiple secondary metabolites. These secondary metabolites play a crucial role in regulating a wide range of plant traits. In current studies, researchers simply combined several light qualities and concluded the effect of a particular light quality. This approach was not objective, and it overlooked the combined effect of multiple light qualities on the plant. Due to the complexity of light qualities and plant traits, we introduced Spearman’s rank correlation coefficient, which used the magnitude of the correlation coefficient to establish a link between complex light qualities and multiple plant traits. Furthermore, to explore the future changes of plant traits under different light qualities, we used the Informer model after CEEDMAN preprocessing to make predictions. The CEEDMAN method preprocessing provided diversity to the data and effectively avoided overfitting of the prediction model, significantly improving the prediction accuracy of the model. Manual intervention of the model, as inferred from the correlation coefficients, also notably improved the overall prediction. The model proposed in this study achieved the best combined performance in both evaluation metrics, $R^{2}$ and SEP .

In the actual planting and production process, crops might be cultivated for various purposes, and growers could adjust the light quality according to the current planting purpose, predicting the future trait changes of strawberries under that light quality environment, thus achieving the planting goal more efficiently.

## Figures and Tables

**Table 1:** Definition of the dependent traits.

Definition	Parameters	Acquisition Time
Change of plant height	Change of the maximum height from the soil part to the top of the leaf crown.	Every half day
Change of leaf count	Change in the count of leaves in plant samples.	Every half day
Change of leaf area	Change in the total leaf area in plant samples.	Every half day
Change of stolon	Change in the count of stolons in plant samples.	Every half day
Time of flowering	The number of days since the last harvest (or planting).	When the flowers bloom
Count of fruit	The total count of fruits produced on the sample from the beginning to the end of the experiment.	At the end of experiments of all subgroups
Time of fruit ripeness	The number of days from fruiting to harvest.	After each harvest
TSS content of fruit	Post-harvest measurements with a Brix meter.	After each harvest
Skin hardness of fruit	Post-harvest measurements with a hardness tester.	After each harvest

**Table 2:** The evaluation indicator performance on compared prediction models.

Method	Transformer		Informer		CEEDMAN with Informer		CEEDMAN, Correlation with Informer	
	$R^{2}$	SEP(%)	$R^{2}$	SEP(%)	$R^{2}$	SEP(%)	$R^{2}$	SEP(%)
Plant height	0.5864	52.1653	0.6016	42.1654	0.7213	33.2345	0.7651 *	28.2684 *
Leaf count	0.4675	60.3543	0.5075	54.3246	0.6854	46.7570	0.8433 *	24.7163 *
Leaf area	0.4035	64.1643	0.6324	46.1634	0.6574	40.1356	0.6831 *	31.1538 *
Stolon count	0.3785	72.5463	0.4237 *	70.3154	0.4169	66.1395 *	0.4094	68.1543
Time of flowering	0.4267	67.3642	0.5079	56.6237	0.4865	45.3785	0.5234 *	37.3623 *
Count of fruit	0.5735	44.7652	0.6095	46.6340	0.6842 *	40.2334	0.6796	34.3266 *
Fruit ripeness	0.5304	54.1748	0.4571	63.5465	0.5903 *	42.6349 *	0.5876	44.3160
TSS content	0.6264	31.1025	0.6234	32.3156	0.6762	28.7831 *	0.6832 *	29.1359
Skin hardness	0.3242	85.3152	0.4529 *	69.3215 *	0.4216	76.3165	0.4463	73.3152

Note: The optimal indicators are labeled with *.

**Table 3:** Key hyper-parameters in proposed model.

Parameter	Value	Parameter	Value
Batch size	128	Dropout	0.05
Epochs	50	Loss function	MSE
Leaning rate	$4\times 10^{-4}$	Activation	GeLU

## Data Availability

Data are available upon request.
